# Rapid and Successful Treatment of Generalized Granuloma Annulare With Deucravacitinib

**DOI:** 10.7759/cureus.98761

**Published:** 2025-12-08

**Authors:** Michael J Lee, Maria Isabelle P Avenido, Emily Ames, David G Cotter

**Affiliations:** 1 Medicine, University of Nevada Las Vegas School of Medicine, Las Vegas, USA; 2 Dermatology, University of Nevada Las Vegas School of Medicine, Las Vegas, USA

**Keywords:** aphthous ulcers, deucravacitinib, granuloma annulare, immuno-modulator, interstitial granuloma annulare, selective tyk2 inhibitor

## Abstract

Granuloma annulare (GA) is a chronic granulomatous dermatosis that often proves resistant to conventional therapies. Increasing evidence implicates interferon-gamma-mediated JAK-STAT activation in GA pathogenesis, with tyrosine kinase 2 (TYK2) playing a central role through IL-12, IL-23, and type I interferon signaling. Deucravacitinib, a selective allosteric TYK2 inhibitor, has emerged as a potential therapy by targeting these pathways. We present the case of a 59-year-old woman with biopsy-confirmed interstitial-type GA that was unresponsive to topical corticosteroids. Initiation of deucravacitinib 6 mg daily led to complete clearance of lesions within one month. Reported adverse effects were limited to brief episodes of aphthous ulcers during the first two weeks of therapy, which resolved spontaneously. Laboratory testing two weeks after treatment initiation showed mild elevations in lipids and glucose and slightly decreased calcium, though baseline labs were not available for comparison and no serious adverse events occurred. This case highlights the potential of deucravacitinib as a novel therapeutic option for refractory GA and adds to emerging evidence that TYK2 inhibition may represent a targeted and effective approach for this condition.

## Introduction

Granuloma annulare (GA) is a chronic, granulomatous skin condition that presents with annular papules or plaques, often resistant to standard treatments, with an incidence of 0.04% and a prevalence of 0.06% in the United States [1-3]. While its etiology remains uncertain, recent evidence points to a dysregulated immune response involving Th1-mediated interferon-gamma (IFN-γ) signaling and downstream JAK-STAT pathway activation [1]. Histologically, GA shows palisading or interstitial histiocytes surrounding collagen with mucin deposition [2]. Although spontaneous remission can occur, many patients experience persistent or generalized disease that proves resistant to conventional therapies such as corticosteroids, retinoids, or systemic immunosuppressants [1,4]. Granuloma annulare has also been associated with metabolic comorbidities, particularly dyslipidemia and diabetes mellitus, most frequently in generalized forms of the disease [2,5].

Advances in immunology have reshaped the understanding of GA pathogenesis. Lesional tissue demonstrates enrichment of Th1 cytokine pathways, particularly IFN-γ, with downstream JAK-STAT activation [1,4]. This framework has driven interest in targeted inhibition of cytokine signaling. Recent clinical reports show improvement of GA with JAK inhibitors, delivered both systemically and topically, highlighting this pathway as a promising therapeutic target [6-8]

Tyrosine kinase 2 (TYK2), a member of the JAK family, selectively mediates signaling through IL-12, IL-23, and type I interferons [9]. Genetic data in humans show that TYK2 deficiency impairs IL-23-dependent IFN-γ responses, underscoring its central role in granulomatous immunity [10]. Deucravacitinib selectively binds the regulatory pseudokinase (JH2) domain of TYK2, allosterically modulating TYK2 and thus attenuating these pro-inflammatory pathways while sparing JAK2-dependent hematopoiesis, potentially offering greater selectivity and safety compared to broader JAK inhibition [11]. Early clinical experiences, including a recent case of generalized GA successfully treated with deucravacitinib, suggest therapeutic potential in this setting [12].

Here, we present a case of interstitial GA refractory to topical corticosteroids that achieved rapid, complete clearance with deucravacitinib.

## Case presentation

**Figure 1:**
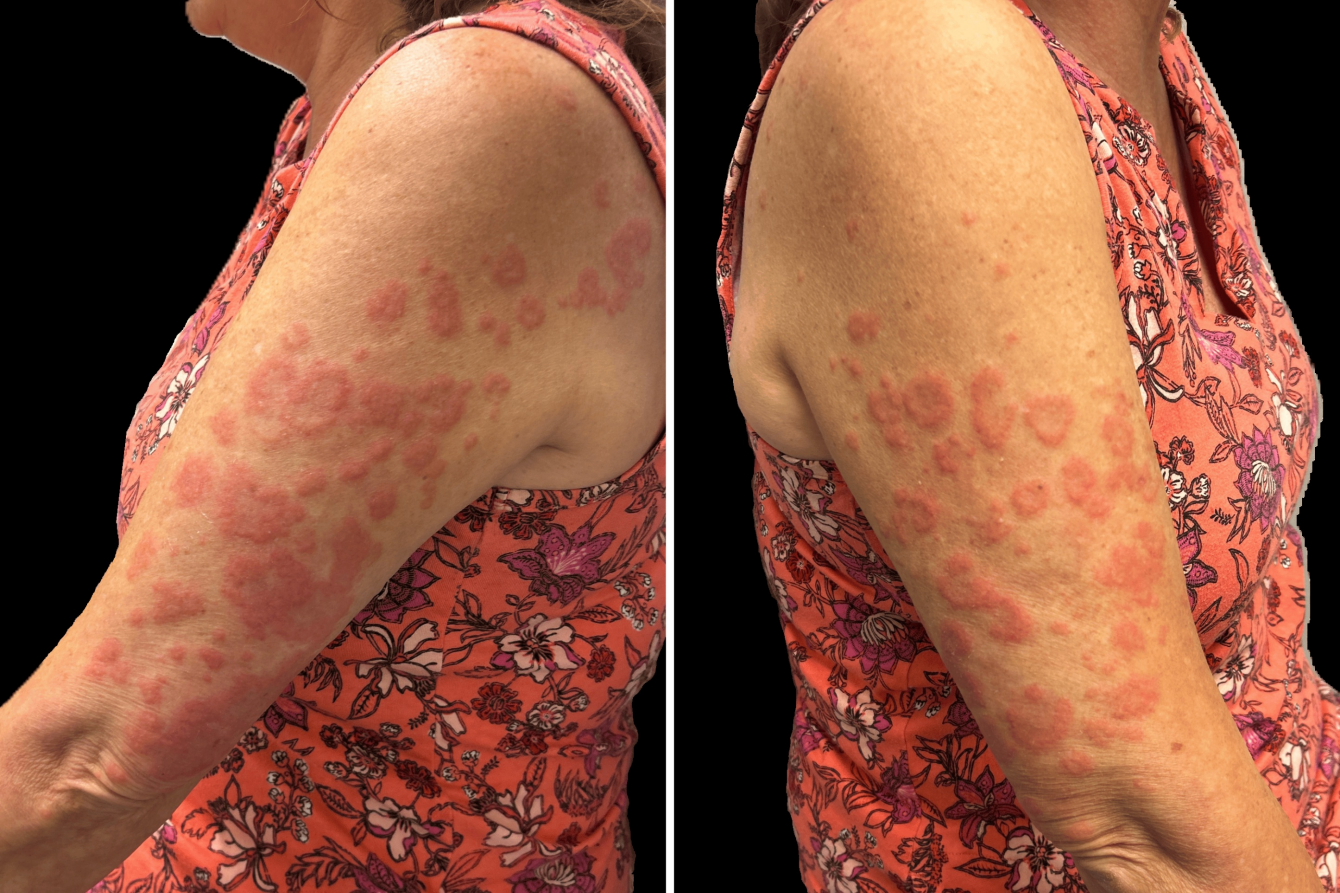
Annular pink, erythematous plaques without scale, consistent with granuloma annulare, present on both arms at the patient’s initial visit.

A 59‑year‑old woman presented with a one‑year history of firm, annular, pink and erythematous plaques on her arms and legs (Figure 1). A biopsy from the left posterior axillary skin revealed interstitial‑type GA. She was initially treated with clobetasol ointment without improvement and subsequently began deucravacitinib 6 mg daily.

Baseline metabolic laboratory studies were not obtained prior to therapy. Laboratory testing performed approximately two weeks after treatment initiation demonstrated mildly elevated triglycerides and glucose and slightly decreased calcium. The patient had no personal history of diabetes or dyslipidemia; however, family history was notable for diabetes mellitus, hypercholesterolemia, and heart disease. Without pre‑treatment laboratory data, it is unclear whether these abnormalities represented unrecognized baseline variation, underlying metabolic predisposition, or medication-related adverse effects. No intervention was required, and her primary care provider continued routine monitoring.

**Figure 2:**
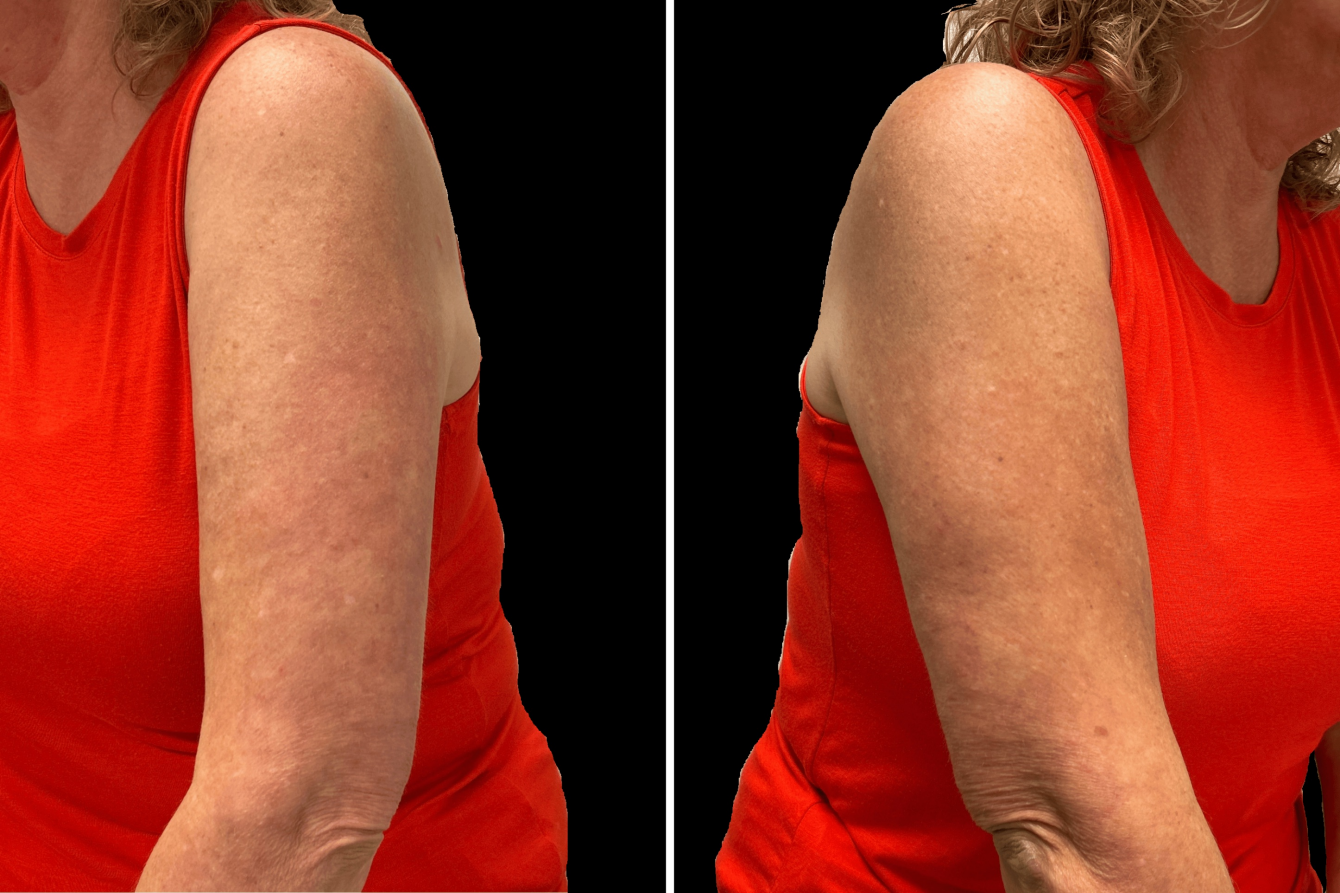
Clear skin showing complete visible resolution of granuloma annulare following one month of deucravacitinib 6 mg daily oral therapy. Images represent the same anatomical sites depicted in Figure 1 under comparable lighting.

The patient reported three brief episodes of aphthous ulcers within the first one to two weeks of therapy, which resolved spontaneously. Her GA skin lesions had completely cleared one month post-deucravacitinib initiation (Figure 2).

## Discussion

This case highlights rapid and complete improvement of refractory interstitial GA with deucravacitinib, consistent with emerging data supporting the role of JAK/TYK2 inhibition in GA management. Traditional therapies, including corticosteroids, retinoids, and antimalarials, have variable and often unpredictable efficacy with frequent relapse [1,2]. In contrast, targeted cytokine blockade, like deucravacitinib, offers a mechanistically informed strategy. 

This outcome aligns with recent reports demonstrating the therapeutic potential of JAK/TYK2 pathway blockade in GA. Evidence indicates that systemic JAK inhibition can induce remission through suppression of IFN-γ-driven inflammatory signaling pathways. Additional reports show consistent benefit across different formulations: topical ruxolitinib cream provided complete clearance in refractory generalized GA, and oral tofacitinib induced remission in otherwise treatment-resistant cases [6-8]. Our patient’s response parallels the case described by Lapa and Breslavets, where generalized GA resolved with deucravacitinib [12], suggesting that TYK2 inhibition may achieve outcomes comparable to broader JAK inhibition.

The consistency of these clinical observations is supported by advances in GA immunopathogenesis. GA is now recognized as a Th1-skewed granulomatous disease driven by IFN-γ and downstream JAK-STAT activation [1,4]. TYK2, in particular, mediates IL-12, IL-23, and type I interferon signaling pathways central to granuloma formation and persistence [9]. Evidence from human TYK2 deficiency further reinforces this role. Patients with inherited TYK2 mutations show impaired IL-23-dependent IFN-γ production and susceptibility to mycobacterial infections, directly implicating TYK2 in granulomatous immunity [10]. Deucravacitinib, as a selective allosteric TYK2 inhibitor, thus offers a mechanistically precise therapeutic strategy.

Adverse effects in our patient were limited to aphthous ulcers, which resolved spontaneously. Laboratory abnormalities have also been reported with JAK inhibitors and highlight the importance of ongoing monitoring during treatment, although such events are infrequent in patients treated with deucravacitinib [13]. However, interpretation of our patient’s metabolic laboratory abnormalities is limited by the absence of pre‑treatment baseline values. Given the known association between GA and metabolic comorbidities, as well as her family history of diabetes and hypercholesterolemia, the mild elevations observed may reflect underlying predisposition rather than a medication effect. Importantly, no clinically significant adverse events occurred, and no treatment was required. This case underscores the importance of obtaining baseline metabolic laboratory testing when initiating TYK2 inhibitors to better contextualize follow‑up results.

A limitation of the current literature is that all reported GA cases treated with deucravacitinib, including this case report, documented favorable outcomes. Treatment failures have yet to be published, which may reflect the novelty of the therapy and possible publication bias. Without systematic data, it is unclear what proportion of patients will respond to the treatment, how durable responses will be, or whether relapse occurs after discontinuation. Therefore, prospective studies are needed to clarify efficacy, durability, long-term safety, and optimal treatment duration.

In summary, this case demonstrates successful treatment of interstitial GA with deucravacitinib after failure of topical corticosteroids. Together with emerging reports, it supports TYK2 inhibition as a targeted and potentially safer alternative to broader JAK blockade. Further systematic study will be essential to establish the role of deucravacitinib in the management.

## Conclusions

This case highlights the rapid clearance of interstitial granuloma annulare with deucravacitinib after inadequate response to topical corticosteroids, adding to growing evidence that TYK2 inhibition is a mechanistically targeted therapeutic option for refractory disease. By addressing cytokine signaling pathways central to granuloma formation, deucravacitinib may offer a more precise strategy than traditional therapies, which often have unpredictable efficacy and high relapse rates.

Although outcomes from early case reports are consistently favorable, systematic studies are needed to define the true efficacy, durability of remission, and relapse risk after discontinuation. Prospective evaluation will be essential to determine the role of TYK2 inhibition in the long-term management of granuloma annulare and to establish its place among available therapeutic options.
